# Bacteriophage inactivation to prevent carry-over in preclinical assays with mycobacteria

**DOI:** 10.21203/rs.3.rs-8650511/v1

**Published:** 2026-01-29

**Authors:** Marta Pozuelo Torres, Carl Morrow, Carlos Andrés Guerrero-Bustamante, Graham F. Hatfull, Andreas H. Diacon, Jakko van Ingen, Saskia Janssen

**Affiliations:** Radboud University Medical Centre, Radboudumc Community for Infectious Diseases & Research Institute of Medical Innovation; TASK; University of Pittsburgh; University of Pittsburgh; TASK; Radboud University Medical Centre, Radboudumc Community for Infectious Diseases & Research Institute of Medical Innovation; Radboud University Medical Centre, Radboudumc Community for Infectious Diseases & Research Institute of Medical Innovation

**Keywords:** Mycobacteriophages, carry-over, phage therapy, mycobacteria

## Abstract

**Background:**

Bacteriophage carry-over, the continued lytic activity of residual phages after experimental sampling, poses a significant challenge in bacteriophage research, potentially skewing experimental results, particularly in the context of preclinical studies where perceived laboratory success may not translate clinically. These inaccuracies, relevant across various bacterial species, underscore the necessity for robust mitigation strategies. This study provides an overview of strategies to mitigate phage carry-over, evaluating their performance on a combination of three mycobacteriophages with lytic activity against *M. tuberculosis*.

**Results:**

We investigated three inactivation methods, including ferrous ammonium sulfate (FAS) and citrate for *in vitro* applications and N-acetyl-L-cysteine-sodium hydroxide (NALC-NaOH) for sputum decontamination. The citrate buffer offered limited and specific phage inactivation, but FAS was effective in inactivating all three mycobacteriophages (FionnbharthΔ45Δ47, Muddy HRM^N0157^-2, and Fred313cpm-1Δ33). With pH adapted to 5.0, FAS did not significantly reduce *M. tuberculosis* H37Rv viability after five minutes exposure. Phage carry-over was determined to be phage-dependent; FionnbharthΔ45Δ47 exhibited the most significant reduction in *M. smegmatis* colony forming units without phage inactivation measures. Furthermore, we showed that NALC-NaOH methods, commonly used for sputum decontamination, are highly effective in reducing phage titers in aqueous solutions and human sputum samples.

**Conclusions:**

This study demonstrates the heterogenous and phage-dependent efficacy of available inactivation buffers, underscoring the critical necessity for researchers to empirically screen their specific phage isolates against chosen inactivation methods. This is crucial to ensure that observed antibacterial effects are correctly attributed to phage activity during the experimental period, rather than to uncontrolled phage carry-over, thereby enhancing the reliability and interpretability of results.

## Background

The emergence and spread of *Mycobacterium tuberculosis* (*Mtb)* and nontuberculous mycobacterial disease with resistance to an increasing number of antibiotics has sparked renewed interest in phage therapy [1,2,3]. Phage therapy represents a novel strategy to combat mycobacterial infections, offering potential advantages over traditional antibiotics. *Mtb*’s low genetic diversity enables predefined phage combinations to treat most patients [1], unlike other fields where larger host diversity exists, requiring individualized combinations.

This resurgence in interest in phage-based research and therapy calls for a critical appraisal of *in vitro* methods to assess phage activity. A critical obstacle herein is phage carry-over: the unintended persistence of phages in samples taken from *in vitro* or *in vivo* models, allowing for uncontrolled further killing of target bacteria after sampling. This phenomenon arises from the persistence of phages in various forms: as free virions in the medium, adsorbed to host cell surfaces, or even internalized within host cells. The uncontrolled phage survival and activity in samples can lead to an overestimation of phage efficacy and hinder reproducibility, particularly in assays studying mycobacteria in physiological states with impaired susceptibility to phage [4,5]. This over-read can generate misleading results, potentially leading to the development of therapies that appear promising in the lab but fail in clinical settings.

While the need for phage inactivation has been explored in various contexts [6,7,8], a standardized method to test and prevent phage carry-over across diverse experimental contexts for mycobacteria is still lacking.

The primary goal of our study was to compare three commonly used inactivation strategies to prevent phage carry-over, including ferrous ammonium sulfate, sodium hydroxide, and citric acid [9,10], focusing on preclinical experiments assessing phage activity against mycobacteria.

## Methods

### Mycobacterial isolates and mycobacteriophages

*M. smegmatis* mc^2^155 was donated by Edith Houben, Amsterdam University Medical Centers and used for phage propagation and quantification in soft agar overlays [11]. *M. smegmatis* mc^2^155 was transferred into Middlebrook 7H9 (M7H9) medium (BD Difco, USA) supplemented with 10% oleic acid-bovine albumin-dextrose-catalase (OADC) (BD, USA) growth supplement and 1mM calcium chloride (CaCl2, Sigma-Aldrich, USA) prior to use for soft agar overlays. Tween 80 (0.05%) was used in starter cultures but omitted in subcultures used for phage infections [12,13].

*Mtb* H37Rv (ATCC, 27294, USA) was grown in M7H9 media supplemented with 10% OADC containing 0.05% Tween 80 at 37°C for 5 days until mid-log phase was reached.

Three mycobacteriophages, namely Fionnbharth (FionnbharthΔ45Δ47), Muddy (Muddy HRM^N0157^-2) and Fred313 (Fred313cpm-1Δ33) were selected from a five-phage combination [1] based on their lytic activity against most *Mtb* lineages and favourable manufacturing properties. All phages were originally isolated on *M. smegmatis* mc^2^ 155 in the context of the SEA-PHAGES teaching programme [14]. The phages were propagated on lawns of *M. smegmatis* mc^2^ 155 and high-titer lysates were prepared with polyethylene glycol precipitation and purified with ultrafiltration as described before [4]. Fred313 was further processed by harvesting the high-titer lysates from agar overlays and passed through 0.22 micron filters [15].

### Sputum samples

Overnight sputum was collected from adult patients with pulmonary TB at TASK clinical trials unit (Cape Town, South Africa), in the context of a study evaluating activity of phage against mycobacteria in sputum.

### Phage and mycobacterial quantification

Phage quantitation was done by spot testing, following an established protocol, by spotting 3 μl of serial dilutions on a *M. smegmatis* lawn [16]. For quantification of *Mtb* H37Rv and *M. smegmatis* colony forming units (CFUs), serial dilutions of bacterial suspensions were prepared and 10 μl spotted in triplicate onto Middlebrook 7H11 and 7H10 agar plates, respectively. Plates were incubated at 37°C, and colonies were counted after 3 weeks or two days, respectively.

### Comparison of efficacy of phage inactivation buffers

Aliquots of 30μL of phage lysates from Fionnbharth, Muddy, and Fred313 were combined with an equal volume of phage buffer (10 mM Tris pH 7.5, 10 mM MgSO4, 68.5 mM NaCl, and 1 mM CaCl_2_) as a negative control, citrate phage inactivation buffer (40 mM Citric acid and 10 mM Potassium chloride at pH 3), 20 mM FAS at pH 5 (Sigma-Aldrich, USA) and 50 mM Bis-Tris + 20 mM FAS at pH 6.5, respectively. Immediately after combining the solutions, a 10 μL sample was ten-fold serially diluted from 10^−1^ to 10^−8^. After a 15-minute incubation period following the initial combination of phage lysate and buffer, another 10 μL sample was taken and serially diluted. Phage titers were quantified on *M. smegmatis* lawns as described above.

### Determination of FAS pH effect on Mtb H37Rv

The efficacy of FAS at pH 5.0 was compared to FAS at pH 6.5 for minimizing potential negative effects on bacterial viability. *Mtb* H37Rv culture was pelleted (5 min, 5,000 × g, RT), washed twice in phosphate buffered saline (PBS) containing Tween 80 (0.05%), needle-passaged and resuspended in PBS before being adjusted to 0.5 McFarland (4.5 × 10^7^ CFU/mL). Inocula were standardized to 1 × 10^6^ CFU per well. Assays were conducted in 96-well plates (200 μL final volume). Each well received the bacterial suspension in M7H9 medium, and 100 μL buffer solution (final treatments: M7H9 control/RPMI control, 50 mM Bis-Tris pH 6.5, 20 mM FAS pH 5.0, or 50 mM Bis-Tris + 20 mM FAS pH 6.5). Bacteria were exposed for 0 or 15 min at room temperature before quantification of CFU as described above.

### Assessment of phage specific carry-over and performance of FAS with M. smegmatis

Phage suspensions were prepared by adding each phage into glass bottles containing 10 mL of M7H9, OADC and CaCl_2_ at a final multiplicity of infection (MOI) of 1: 10 (bacterium:bacteriophage). Bacterial inoculation was performed with *M. smegmatis* to reach a final concentration of 1 × 10^6^ CFU/mL. Samples were collected for bacterial and phage quantification; Fred313 and Fionnbharth were sampled on days 1 and 2 while Muddy was sampled on days 2 and 3 to account for its slower replication characteristics. Prior to sampling, bottles were vortexed and thoroughly mixed before extracting 1 mL aliquots for each condition: untreated, FAS-treated (pH 5), and supernatant discarded. For the FAS-treated and supernatant discarded conditions, samples were centrifuged at 8,000 rpm for 5 minutes. The supernatant was collected and analyzed for PFU presence in phage buffer. Subsequently, pellets were resuspended in 1 mL of either FAS or M7H9 (Tween-free) accordingly, incubated for 5 minutes, then subjected to a second centrifugation. Pellets were resuspended in M7H9 supplemented with Tween. Finally, 100 μL aliquots from each sample were transferred to a 96-well plate and serially diluted in saline for quantification of CFUs.

### Phage inactivation with sputum decontamination

To investigate the effectiveness of sodium hydroxide (NaOH) in deactivating phages, two routinely applied standard decontamination methods were employed: NALC-NaOH (NAC-PAC^™^ Red, AlphaTec, USA) and NALC-NaOH (MycoPrep^™^ system, BD BBL). Experiments were conducted with aqueous phage solutions and human sputum samples. Aqueous phage solutions of the three individual phages and their combination were treated according to the outlined decontamination protocols. After treatment, the titre of the phage was determined. Two samples of human sputum were incubated with the three-phage combination for 24 or 48 hours at 37°C. Following incubation, all samples were decontaminated and then filtered through a 0.22 μm syringe filter to obtain sterile supernatant for phage quantification.

## Results

### Comparison of efficacy of phage inactivation buffers and determination of FAS effect on Mtb H37Rv

**Figure 1:**
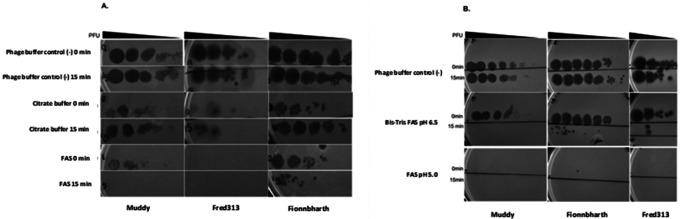
Comparison of efficacy of different phage inactivation buffers A) Comparison of citrate buffer (40mM citric acid, 10mM potassium chloride, pH 3) versus ferrous ammonium sulfate (20mM FAS, pH 5) inactivation on *M. smegmatis*, after 0 and 15 minutes of exposure. B) Comparison of FAS inactivation buffer at pH 5 versus pH 6.5 on *M. smegmatis*, after 0 and 15 minutes of exposure.

When comparing the performance for *in vitro* phage inactivation of FAS and citrate buffer, results indicated that FAS had a broader effect in inactivating all three phages tested (Fig. 1A). Moreover, 15 minutes of incubation with the FAS inactivation buffer showed bigger drops in titer than with no incubation (Fig. 1A). The citrate buffer demonstrated efficacy specifically against Fred313 but was less effective in reducing titers of Muddy and Fionnbharth.

FAS at pH 5.0 was the most effective in visibly reducing phage titers (Fig. 1B). However, the buffered FAS at pH 6.5 achieved similar inactivation results after a 15-minute incubation period (Fig. 1B), suggesting a potential alternative for applications where a less acidic environment is preferred or necessary for bacterial integrity.

**Figure 2:**
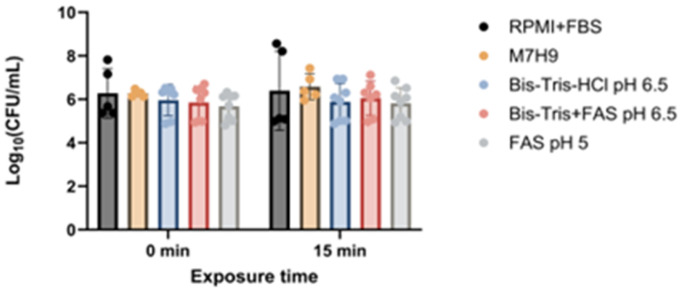
Effect of different pH phage inactivation buffers on *Mtb H37Rv*. The effect was measured after 0 and 15 minutes of exposure to the inactivation buffer (n=3). Note: none of the comparisons showed significant statistical difference.

Across all tested conditions, none of the buffer formulations significantly reduced *Mtb* H37Rv growth compared with the media control (Fig. 2).

### Assessment of phage specific carry-over and performance of FAS with M. smegmatis

**Figure 3:**
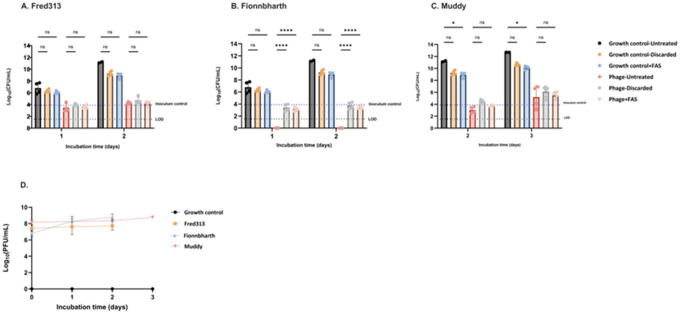
Assessment of phage specific carry-over with and without FAS treatment. A-C) Mycobacterial load survival in Log_10_(CFU/mL). Graphs A, B, and C represent data for Fred313, Fionnbharth, and Muddy, respectively. Fred313 and Fionnbharth were sampled on days 1 and 2, while Muddy was sampled on days 2 and 3. The blue dotted line represents the inoculum control, indicating the starting amount of *M. smegmatis*. The black dotted line represents the limit of detection (LOD). Data was analyzed via two-way ANOVA with a Tukey post-hoc test (n=2; ns: non-significant, *p ≤ 0.05, **p ≤ 0.01, ***p ≤ 0.0001). D) Phage concentrations in Log_10_(PFU/mL).

The effectiveness of different strategies, FAS treatment and discarding of supernatant, in mitigating phage carry-over and their subsequent impact on *M. smegmatis* bacterial load was investigated in a time-kill experiment. Concentrations of phages between 10^6^ and 10^8^ PFU/mL were consistently detected in the supernatants of infected cultures throughout the 72-hour period (Fig. 3D), indicating phage replication and persistence in the culture medium.

In the growth control groups, *M. smegmatis* consistently maintained high viability, with CFU counts increasing over time. However, when the bacterial density increased during days 2 and 3, a bactericidal effect was observed in the growth control conditions with phage inactivation strategies resulting in 2 log reduction in CFUs.

Phage carry-over was phage-dependent and most pronounced for Fionnbharth (Fig. 3B) which exhibited the most significant reduction in CFU without phage inactivation measures, with CFUs below the detection limit after 24 hours. FAS treatment as well as removal of phage containing supernatant adequately prevented phage carry-over.

### Effectiveness of sputum decontamination for phage inactivation

Both decontamination methods demonstrated significant reduction in phage titres, often leading to undetectable levels of phage (Table 1).

After 24 or 48 hours of sputum incubation with phages at an estimated concentration of 10^9^ pfu/mL and subsequent decontamination, phage titres in filtered sputum supernatants were significantly reduced, often below the limit of detection (Table 2). Overall, both NaOH decontamination treatments were highly effective at reducing phage activity in sputum.

## Discussion

Phage carry-over represents a significant caveat in bacteriophage research, influencing the accuracy of preclinical experimental results and hindering their reproducibility. Our study provides an overview of strategies to mitigate phage carry-over, with a specific focus on assays involving mycobacteria.

The different inactivation buffers present distinct advantages and disadvantages. Citrate buffer demonstrated limited and phage dependent efficacy in reducing the phage titers. In contrast, FAS inactivated all three phages tested and was previously shown to inactivate mycobacteriophage D29 [17]. The mechanism of action is poorly understood but might involve oxidative mechanisms that damage phage components [18]. However, without buffering the pH of FAS is around 4, carrying potential for metabolic and genetic shifts [19]. When buffered, the pH is instable thus requiring immediate use after preparation. While FAS at pH 5.0 was most effective at reducing phage titers, buffered FAS at pH 6.5 achieved similar inactivation after a 15-minute incubation, offering a less acidic alternative. Crucially, our evaluation of FAS’s effect on *Mtb* H37Rv revealed no significant reduction in bacterial growth across tested pH values, suggesting that FAS primarily acts on the phage without substantial direct adverse effects on the mycobacterial host. Our findings from the time-kill assay underscored the phage-specific carry-over. This highlights the critical need for researchers to investigate the most suitable inactivation method before engaging in more complex infection experiments [9].

For phage inactivation in sputum samples, our results showed that routinely used NALC-NaOH methods achieved significant reductions in phage titers. This alleviates the need for a dedicated phage inactivation step in future clinical studies assessing mycobacterial load in sputum if NALC-NaOH is part of the protocol, provided inactivating effect is confirmed on the phage used.

Our study limitations include that only three phages were examined, which restricts generalizability of the findings. In the time-kill assay, we exclusively evaluated the efficacy of FAS, not extending this assessment to other methods like NaOH or citric acid. Finally, for sputum samples, the absence of a baseline phage titer for untreated sputum samples precluded an exact assessment of phage titer reduction.

## Conclusions

This study significantly advances phage research by demonstrating the phage-specific efficacy of inactivation buffers and the critical need for pre-validation. The data shows that while citrate buffer had limited effect, FAS at pH 5.0 effectively inactivated the three tested phages without harming *Mtb* H37Rv growth. We also found that routine NALC-NaOH methods effectively reduce phage titers in sputum samples, potentially obviating a dedicated inactivation step in clinical studies. These findings provide practical, evidence-based strategies for researchers to enhance experimental integrity and reproducibility, thereby supporting the advancement of phage therapy.

## Figures and Tables

**Table 1:** Phage inactivation using sodium hydroxide-based decontamination procedures

Phage	Decontamination protocol	PFU/ml; no decontamination	PFU/ml; with decontamination	Log Change
Muddy HRM^N0157^-2	NACPAC	> 1×10^10^	<LOD	> 7.5
	Mycoprep	> 1×10^8^	3.33×10^2^	> 5.5
Fred313cpm-1Δ33	NACPAC	1.93 ×10^7^	<LOD	> 4.8
	Mycoprep	4×10^8^	6.67×10^2^	5.8
FionnbharthΔ45Δ47	NACPAC	> 1×10^10^	1×10^4^	> 6.0
	Mycoprep	2.67×10^8^	6.67×10^2^	5.6
Phage combination	NACPAC	4×10^9^	<LOD	> 7.1
	Mycoprep	1×10^8^	<LOD	> 5.5

<LOD means below the limit of detection which was 333 pfu/mL.

**Table 2:** Phage inactivation by sputum decontamination

Sputum sample	Phage incubation (hrs)	Plaques per μL	Total PFU/mL
T12_034	24	2.67	2667
	48	0	<LOD
T12_044	24	0	<LOD
	48	0	<LOD

<LOD means below the limit of detection which was 333 pfu/mL.

## Data Availability

Data will be made available upon reasonable request.
